# Comparison of Dexmedetomidine and Fentanyl as Adjuvants to Ropivacaine for Epidural Analgesia in Abdominal Hysterectomy: A Study on Postoperative Analgesia Quality

**DOI:** 10.7759/cureus.87155

**Published:** 2025-07-02

**Authors:** Puja Kumari, Mukesh Kumar, Nitesh Sinha, Vishwanath Kumar, Dipali Singh

**Affiliations:** 1 Anesthesiology, Shaheed Nirmal Mahato Medical College and Hospital (SNMMCH), Dhanbad, IND; 2 Anesthesiology, Rajendra Institute of Medical Sciences, Ranchi, IND

**Keywords:** dexmedetomidine, fentanyl, patient-controlled epidural analgesia, ropivacaine, total abdominal hysterectomy

## Abstract

Background and aim

Ropivacaine, at lower concentrations, has been shown to provide effective postoperative analgesia. The addition of adjuvants enhances its analgesic efficacy without significantly increasing the risk of motor blockade. The study aimed to evaluate the analgesic adjuvant properties of epidural dexmedetomidine (1 µg/mL) and epidural fentanyl (2 µg/mL) when administered with 0.125% ropivacaine via a patient-controlled epidural analgesia (PCEA) pump in patients undergoing total abdominal hysterectomy (TAH).

Methods

A total of 60 patients scheduled for elective TAH were randomized into two groups in this prospective trial. Thirty patients were allocated to the ropivacaine with dexmedetomidine group (Group RD), and 30 patients were enrolled in the ropivacaine with fentanyl group (Group RF). Group RD received 0.125% ropivacaine with dexmedetomidine (1 µg/mL) as the study drug, whereas Group RF received 0.125% ropivacaine with fentanyl (2 µg/mL). Patients were administered general anesthesia after placement of a lumbar epidural catheter at the L3-L4 interspace, advanced 3-5 cm into the epidural space. One hour after skin incision, the study drugs were continuously infused through the epidural catheter using a syringe pump at a rate of 7 mL/hour. Following completion of surgery and extubation, PCEA pumps were connected to the epidural catheter, delivering a background infusion of 5 mL/hour, a bolus dose of 2 mL, and a lockout interval of 10 minutes. The background infusion was continued for 24 hours. Rescue analgesia with fentanyl (0.5 µg/kg) was planned if the Visual Analog Scale (VAS) score remained above three despite reaching the maximum permissible PCEA dose of 10 mL/hour. Postoperatively, patients undergoing TAH were monitored for total study drug consumption via the epidural route through PCEA, postoperative pain intensity (measured using VAS), the number of self-administered bolus doses via the PCEA pump, need for rescue analgesics, hemodynamic parameters, and other adverse effects.

Results

VAS pain scores were found to be statistically significant between the two groups at 4, 8, 12, 16, 20, and 24 hours postoperatively, with p-values of 0.034, 0.002, 0.004, 0.001, 0.005, and 0.023, respectively. The total drug consumption during and after surgery was 138.47 ± 2.67 mL for the RD group and 144.53 ± 4.19 mL for the RF group, with a p-value of 0.0001, indicating a highly significant difference. The average total number of bolus doses in 24 hours was 3.067 ± 1.23 in Group RD and 5.267 ± 2.09 in Group RF, with a p-value of 0.0001. Rescue analgesia was not required in either group, and no motor blockade was observed in any patient. Hemodynamic parameters were comparable between groups and did not show any significant changes.

Conclusions

The analgesic adjuvant properties of dexmedetomidine (1 µg/mL) were superior to those of fentanyl (2 µg/mL) when used with 0.125% ropivacaine. However, neither group required rescue analgesia, suggesting that both agents can be effectively used for TAH.

## Introduction

Effective management of postoperative pain is critical in ensuring a smooth recovery process, particularly in major surgical procedures like total abdominal hysterectomy (TAH). Epidural analgesia, when combined with adjuvants, offers a significant advantage by providing superior pain relief and minimizing systemic side effects. Ropivacaine, a commonly used local anesthetic, is favored for its ability to offer effective sensory blockade with minimal motor impairment, making it ideal for postoperative analgesia in lower concentrations [1]. Ropivacaine at 0.125% concentration has been found to be devoid of motor blockade and has been used with adjuvants for prolonging pain relief [2].

Several studies have examined the addition of dexmedetomidine to epidural ropivacaine for pain management, consistently showing that dexmedetomidine enhances the quality of the block by extending its duration and increasing the depth of analgesia [3-5]. Various doses of fentanyl and local anesthetic combinations have been studied for epidural anesthesia in obstetric and non-obstetric patients. These studies have used different combinations of fentanyl and local anesthetics and different parameters to assess the quality of epidural anesthesia [6-8].

In 1988, Gambling et al. introduced "patient-controlled epidural analgesia" (PCEA) for managing pain during labor [9]. This innovative technique empowered patients to adjust the amount of analgesic they received according to their personal pain levels and needs. Initially, PCEA involved only patient-initiated boluses, but over time the practice began to add a continuous background infusion to complement the patient-initiated doses, enhancing overall pain management and patient comfort. This study also uses a basal background infusion followed by patient-administered top-up doses.

The study primarily aimed to compare the efficacy of dexmedetomidine and fentanyl as analgesic adjuvants to ropivacaine for managing postoperative pain in patients undergoing abdominal hysterectomy. Utilizing PCEA, the study evaluated the quality of analgesia by focusing on parameters such as visual analog scale (VAS) pain scores, the number of patients administered doses on the PCEA pump, and total drug consumption via the PCEA pump over a 24-hour period. Given the importance of optimizing pain management protocols in major surgeries, this study provides valuable insights into the comparative effectiveness of these adjuvant combinations, potentially guiding clinical practice in the use of epidural analgesia for abdominal hysterectomy patients.

## Materials and methods

The study was a randomized, double-blind, controlled trial conducted over one year at a tertiary care center in eastern India, the Rajendra Institute of Medical Sciences (RIMS), Ranchi. The study protocol received approval from the Institutional Ethics Committee of RIMS, Ranchi (vide memo no. 101 dated 12/04/21), and was registered with the Clinical Trials Registry, India, under registration number CTRI/2022/01/039726. Patients aged 30 to 60 years with American Society of Anesthesiologists (ASA) physical status I or II, scheduled for abdominal hysterectomy and who provided written informed consent, were included in the study. Patients unwilling to provide written informed consent, those with known allergies to the study drugs, or patients with bleeding diathesis, uncontrolled hypertension, abnormal heart rates, or local neuraxial infections were excluded from the study.

Of the 63 patients assessed for eligibility, three declined to provide consent due to concerns about the type of intervention and were therefore excluded from the study. The remaining 60 patients were enrolled and randomized into two equal groups using computer-generated numbers. Allocation concealment was ensured through opaque, sealed envelopes. Group RD received ropivacaine combined with dexmedetomidine for analgesia, while Group RF received ropivacaine combined with fentanyl. Blinding was maintained by concealing drug allocation from both the patients and the observer assessing analgesia. The drugs were administered by an anesthetist who was unaware of the specific study drugs used. The study protocol involved two randomized groups receiving different drugs, as mentioned above, with similar infusion regimens. In Group RD, ropivacaine (0.125%) combined with 1 µg/mL dexmedetomidine was infused at 7 mL/hr starting 1 hour after skin incision. This rate was reduced to 5 mL/hr after tracheal extubation and continued for 24 hours postoperatively. In Group RF, ropivacaine (0.125%) combined with 2 µg/mL fentanyl was infused at the same initial rate of 7 mL/hr 1 hour after skin incision, then reduced to 5 mL/hr after tracheal extubation and continued for 24 hours postoperatively.

All patients underwent a thorough pre-anesthetic evaluation, which included a comprehensive medical history, general physical examination, and systemic assessment. The study's purpose and nature were clearly explained to the patients who qualified for enrollment, and written informed consent was obtained after their prior verbal approval.

Patients were instructed to fast for 8 hours prior to surgery. Any anxiety or apprehension was managed with 0.25 mg of alprazolam the night before surgery. Upon arrival in the preoperative room, intravenous access was secured in the non-dominant forearm using a 20G intravenous cannula. Routine premedication, including 50 mg of ranitidine and 10 mg of metoclopramide, was administered 30 minutes before the induction of anesthesia.

In the operating room, standard monitoring devices were used, including ECG, non-invasive blood pressure (NIBP), and pulse oximetry, and baseline measurements were recorded. An epidural catheter was placed at the L3-L4 interspace with the patient in a sitting position using an 18G Tuohy needle. The epidural space was identified using the loss of resistance technique. A test dose of 3 mL of 2% lignocaine with adrenaline was administered, and after confirming correct placement, the catheter was secured with 3-5 cm of its length inside the epidural space. The patient was then positioned supine. Heart rate, mean arterial pressure (MAP), and SpO₂ were recorded before and after administration of the epidural test dose. General anesthesia was induced with propofol (2 mg/kg), vecuronium (0.1 mg/kg), and fentanyl (2 µg/kg), and maintained with a gas mixture of oxygen, nitrous oxide, and isoflurane. Muscle relaxation was maintained with intermittent boluses of vecuronium (0.02 mg/kg). One hour after skin incision, the respective study drugs were infused via the epidural catheter using a syringe pump. Group RD received 0.125% ropivacaine with 1 µg/mL dexmedetomidine at a rate of 7 mL/hour, while Group RF received 0.125% ropivacaine with 2 µg/mL fentanyl at the same rate. At the end of surgery, patients were reversed from anesthesia and their tracheas were extubated.

Patients received postoperative analgesia via PCEA using the Fresenius Kabi Agilia SP PCEA pump, with the same drug combinations as those used intraoperatively. The postoperative analgesia protocol for both groups included a background infusion at 5 mL/hour, a demand bolus of 2 mL, and a lockout interval of 10 minutes, with a maximum allowable dose of 10 mL per hour. Pain was assessed using the VAS. A VAS score exceeding three, despite the maximum permissible dose of 10 mL/hour, was considered indicative of breakthrough pain. Rescue analgesia for breakthrough pain consisted of a bolus of fentanyl (0.5 µg/kg).

Observations included continuous monitoring of VAS pain scores at 0, 1, 2, 4, 8, 12, 18, and 24 hours post-surgery. The sensory level of the block was assessed using the pinprick method at the same time points. Motor blockade was evaluated using the Modified Bromage Scale, where a score of 0 indicated no motor block with full movement of the hip, knee, and ankle; a score of one indicated the inability to raise the extended leg while maintaining movement at the knee and ankle; a score of two denoted the inability to flex the hip and knee but preserved ankle movement; and a score of three corresponded to complete motor block, with no movement possible at the hip, knee, or ankle. Additionally, data were collected on total drug consumption in each group, the total number of PCEA attempts, rescue analgesic use, hemodynamic status, and any side effects during the first 24 hours after surgery.

Statistical analysis

Statistical analysis was performed using SPSS version 28.0 (IBM SPSS Statistics, Armonk, NY). Categorical data were expressed as frequencies and percentages and compared using the Chi-square test. Quantitative data were reported as mean with standard deviation and compared using the unpaired Student's t-test. The sample size for each group was calculated to be approximately 27.23, rounded to 30, using the formula $n = \frac{[P_1 (1 - P_1) + P_2 (1 - P_2)]}{(P_1 - P_2)^2} \times F$. In this formula, n represents the sample size per group, with P1​ set at 35% (or 0.35) and P2 ​ at 75% (or 0.75). The factor F was determined to be 7.9 for 80% power. The calculation was based on a 5% level of significance and 80% power for the study.

## Results

**Figure 1:**
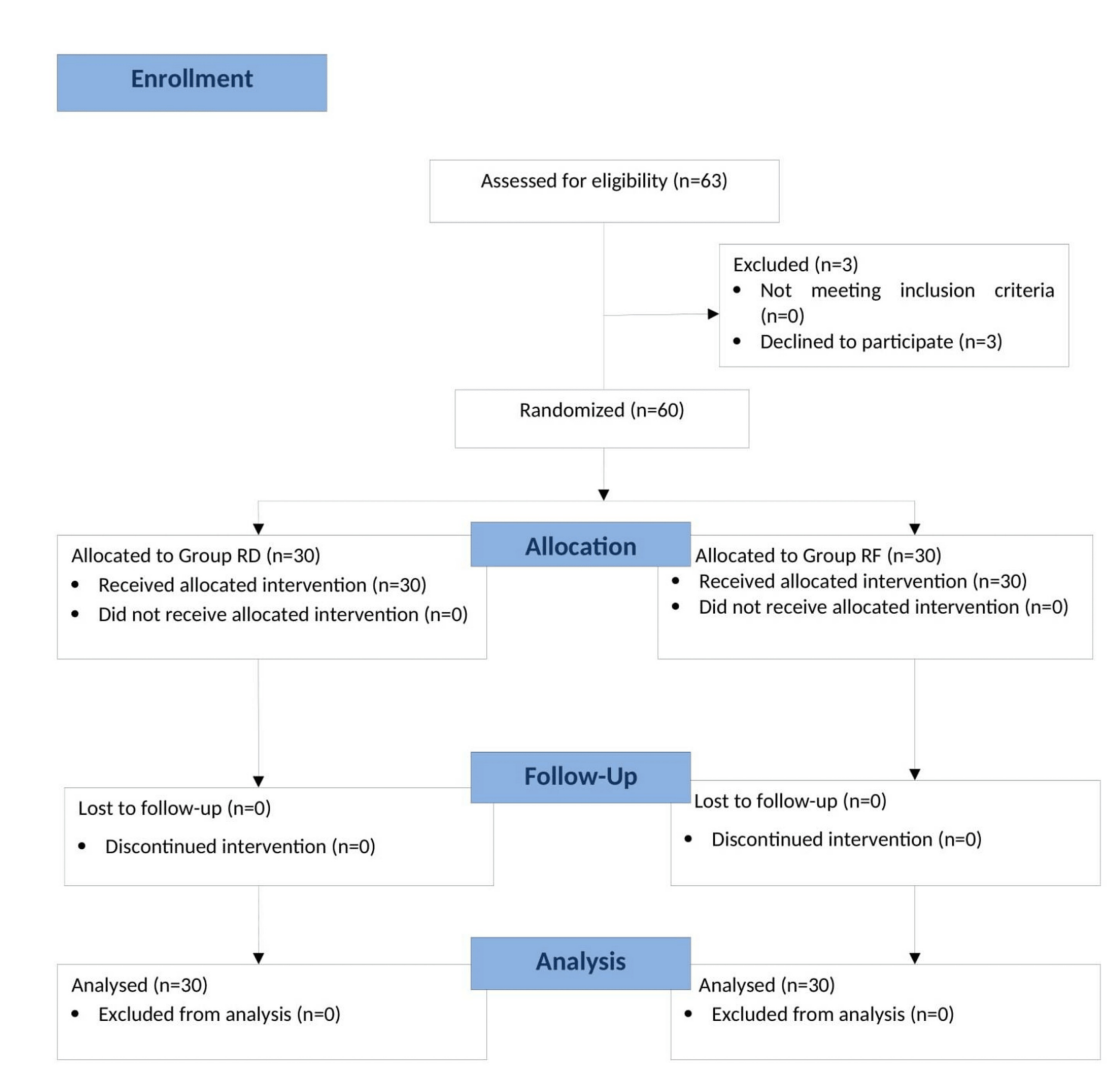
CONSORT flow diagram. CONSORT, Consolidated Standards of Reporting Trials; Group RD, ropivacaine with dexmedetomidine; Group RF, ropivacaine with fentanyl

All 60 enrolled patients completed the study successfully (Figure 1). The demographic variables, including age, weight, height, and surgical duration, showed no statistically significant differences between Group RD and Group RF, indicating that both groups were well-matched for baseline characteristics. Additionally, the distribution of patients according to ASA physical status (Class I and II) was similar, with no significant differences (Table 1).

**Table 1:** Demographic characteristics, surgical data, and ASA classification of the patients. All values were expressed as mean ± SD or number of subjects (%). Comparisons between Group RD and Group RF for continuous variables were made using an independent samples t-test. Comparisons for categorical variables (ASA I and ASA II) were made using the Chi-square test. Group RD, ropivacaine + dexmedetomidine; Group RF, ropivacaine + fentanyl; ASA, American Society of Anesthesiologists

Variable	Group RD (Mean ± SD)	Group RF (Mean ± SD)	Test Statistic	P-value
Age (years)	42.30 ± 5.20	40.33 ± 5.88	t = 1.322	0.184
Weight (kg)	56.97 ± 6.94	56.33 ± 7.13	t = 0.328	0.733
Height (cm)	153.83 ± 5.59	153.20 ± 5.44	t = 0.444	0.663
Surgical duration (minutes)	129.46 ± 12.08	129.40 ± 13.02	t = 0.025	0.981
ASA I	23 (76.66%)	24 (80%)	χ² = 0.342	0.563
ASA II	7 (23.33%)	6 (20%)	χ² = 0.029	0.855

The sensory block reached the T10 dermatome by the end of the second postoperative hour and remained stable at this level across all recorded time points during the first 24-hour postoperative period, with no further cephalad extension or caudal regression. Notably, no motor block was observed at any time during the assessment period.

Pain scores differed significantly between the groups at multiple time points. VAS scores showed a trend of decreasing pain over 24 hours in both groups. Notably, from the 4-hour mark onward, patients in Group RD consistently reported lower pain scores compared to Group RF, with statistically significant differences observed at several time points (4, 8, 12, 16, 20, and 24 hours). This suggests that the analgesic regimen used in Group RD provided superior and sustained pain relief throughout the first 24 hours postoperatively (Table 2).

**Table 2:** VAS scores at different time intervals. All values were expressed as mean ± SD. Comparisons between Group RD and Group RF at each time point were made using an independent samples t-test. Group RD, ropivacaine + dexmedetomidine; Group RF, ropivacaine + fentanyl; VAS, Visual Analog Scale

Time	Group RD (Mean ± SD)	Group RF (Mean ± SD)	Test Statistic	P-value
0 hour	3.300 ± 1.00	3.767 ± 0.88	t = -1.896	0.065
1 hour	2.633 ± 0.79	2.967 ± 0.83	t = -1.549	0.125
2 hour	1.900 ± 0.94	2.233 ± 0.71	t = -1.527	0.135
4 hour	1.433 ± 0.84	1.900 ± 0.79	t = -2.146	0.034
8 hour	1.233 ± 0.84	1.900 ± 0.74	t = -3.074	0.002
12 hour	1.133 ± 0.76	1.700 ± 0.69	t = -3.215	0.004
16 hour	0.933 ± 0.81	1.900 ± 0.83	t = -4.779	0.001
20 hour	0.800 ± 0.79	1.433 ± 0.84	t = -3.468	0.005
24 hour	0.733 ± 0.72	1.200 ± 0.79	t = -2.400	0.023

Group RD not only demonstrated superior pain relief but also showed more efficient use of analgesia. This was evidenced by the significantly lower total drug volume consumed by Group RD compared to Group RF. Additionally, the number of bolus doses administered by patients was significantly lower in Group RD, suggesting better patient satisfaction and a reduced need for additional pain medication in the dexmedetomidine group. Interestingly, no rescue analgesics were required in either group, highlighting the overall effectiveness of both analgesic protocols. However, despite the absence of rescue analgesic use in both groups, Group RF required a higher total drug dose and more bolus administrations to achieve comparable pain relief (Table 3).

**Table 3:** Total drug consumption, PCEA bolus doses, and rescue analgesic use. All values are expressed as mean ± SD. Comparisons between Group RD and Group RF for total drug consumption and number of PCEA bolus doses were performed using an independent samples t-test. Rescue analgesic use was not analyzed, as there were zero occurrences in both groups. Group RD, ropivacaine + dexmedetomidine; Group RF, ropivacaine + fentanyl; PCEA, patient-controlled epidural analgesia

Measurement	Group RD (Mean ± SD)	Group RF (Mean ± SD)	Test Statistic	P-value
Total drug consumption (mL/24 hr)	138.47 ± 2.67	144.53 ± 4.19	t = -6.651	0.0001
No. of PCEA bolus doses in 24 hr	3.067 ± 1.23	5.267 ± 2.09	t = -6.524	0.0001
Rescue analgesic use	0	0	-	-

Regarding hemodynamic variability and stability, both groups demonstrated acceptable outcomes in terms of pulse rate during the first 24 hours postoperatively. Pulse rates remained stable and comparable between the two groups at all measured time points. Although Group RF exhibited slightly higher mean values, none of the differences were statistically significant. These findings suggest that neither regimen adversely affected heart rate and that both maintained acceptable hemodynamic stability (Table 4).

**Table 4:** Postoperative pulse rate at different time intervals. All values were expressed as mean ± SD. Comparisons between Group RD and Group RF at each time point were made using an independent samples t-test. Group RD, ropivacaine + dexmedetomidine; Group RF, ropivacaine + fentanyl

Time	Group RD (Mean ± SD)	Group RF (Mean ± SD)	Test Statistic	P-value
0 hour	80.533 ± 13.990	86.233 ± 14.543	t = -1.430	0.134
1 hour	82.067 ± 12.696	85.133 ± 14.338	t = -0.863	0.392
2 hour	79.733 ± 12.083	83.333 ± 12.880	t = -1.099	0.277
4 hour	79.700 ± 13.335	81.767 ± 12.834	t = -0.604	0.55
8 hour	78.800 ± 12.592	81.033 ± 11.429	t = -0.703	0.482
12 hour	80.800 ± 11.125	80.900 ± 10.995	t = -0.036	0.973
16 hour	80.933 ± 10.289	80.833 ± 10.110	t = 0.025	0.97
20 hour	80.967 ± 9.683	81.067 ± 8.016	t = -0.027	0.966
24 hour	79.733 ± 12.083	80.933 ± 7.882	t = -0.457	0.656

Similar to pulse rate trends, mean blood pressure values remained within a clinically acceptable range throughout the 24-hour postoperative period for both groups. Inter-group differences were minimal and not statistically significant at any time point, further reinforcing the cardiovascular safety of both analgesic strategies (Table 5).

**Table 5:** Postoperative mean blood pressure (mm Hg) at different time intervals. All values were expressed as mean ± SD. Comparisons between Group RD and Group RF at each time point were made using an independent samples t-test. Group RD, ropivacaine + dexmedetomidine; group RF, ropivacaine + fentanyl

Time	Group RD (Mean ± SD)	Group RF (Mean ± SD)	Test Statistic	P-value
0 hour	77.832 ± 5.312	78.533 ± 5.560	t = -0.498	0.619
1 hour	76.800 ± 6.145	78.800 ± 5.576	t = -1.302	0.199
2 hour	77.600 ± 6.075	78.750 ± 5.565	t = -0.780	0.436
4 hour	77.800 ± 6.935	78.400 ± 4.964	t = -0.357	0.706
8 hour	77.620 ± 5.499	78.667 ± 4.422	t = -0.796	0.419
12 hour	78.133 ± 4.410	78.685 ± 5.546	t = -0.474	0.615
16 hour	77.600 ± 4.424	78.667 ± 5.594	t = -0.798	0.424
20 hour	77.200 ± 3.563	78.200 ± 4.542	t = -0.939	0.355
24 hour	77.867 ± 4.759	77.533 ± 4.433	t = 0.286	0.784

The data presented in Table 6 indicate that there were no significant differences in postoperative adverse events between the fentanyl group and the dexmedetomidine group. The rates of respiratory depression, bradycardia, hypotension, oversedation, urinary retention, and pruritus were reported to be none across both groups. Nausea and vomiting were reported in both groups but with p-values exceeding the conventional threshold for significance (p > 0.05).

**Table 6:** Adverse effects noted across the two groups. All values were expressed as the number of subjects reporting the adverse events (%). *Oversedation was defined as a Ramsay Sedation Score of five or more. No statistical comparison was made for adverse events that had zero occurrences in both groups. The p-value for nausea and vomiting was calculated using Fisher’s exact test. Group RD, ropivacaine + dexmedetomidine; group RF, ropivacaine + fentanyl

Adverse Event	Group RD	Group RF	P-value
Respiratory depression	0	0	
Bradycardia	0	0	
Hypotension	0	0	
Oversedation*	0	0	
Pruritus	0	0	
Urinary retention	0	0	
Nausea and vomiting	6 (20%)	9 (30%)	0.157

## Discussion

Both dexmedetomidine and fentanyl, when used as adjuvants to low-concentration ropivacaine (0.125%) in epidural anesthesia, provide effective postoperative analgesia without significant motor blockade. However, dexmedetomidine offers superior analgesic efficacy, characterized by a longer duration of analgesia, lower VAS scores, no need for rescue analgesia, and overall lower drug consumption. These findings are consistent with previous studies and support the consideration of dexmedetomidine as a preferable epidural adjuvant for enhanced postoperative pain management in TAH patients.

This study evaluated the analgesic efficacy of dexmedetomidine and fentanyl as adjuvants to low-concentration ropivacaine (0.125%) in postoperative epidural analgesia for patients undergoing TAH. The epidural catheter was deliberately placed at the L3-L4 interspace to provide effective postoperative analgesia, as intraoperative anesthesia was administered using general anesthesia. This level was selected to ensure optimal segmental coverage of the lower abdominal and pelvic dermatomes (T10-S1), while minimizing the risk of excessive cephalad spread and opioid-related side effects.

The epidural infusion commenced 1 hour after the surgical incision to reduce intraoperative opioid requirements and establish a loading dose. Following extubation, a continuous basal infusion via PCEA enabled precise monitoring of drug consumption and pain control over the first 24 postoperative hours. Our results confirmed that a target sensory block level at T10 was consistently achieved by the end of the second postoperative hour and maintained thereafter.

Previous studies comparing dexmedetomidine and fentanyl with local anesthetics for epidural use have consistently shown superior analgesic outcomes with dexmedetomidine [10-14]. Our findings support this trend, demonstrating that dexmedetomidine was associated with fewer bolus requests, lower total drug use, and better pain scores at various postoperative time points. The use of PCEA in our study allowed precise measurement of drug consumption and patient demand, highlighting the distinct analgesic profile of each adjuvant in a homogeneous population of female TAH patients.

Bajwa et al. reported prolonged analgesia with dexmedetomidine compared to fentanyl in lower limb surgeries using a single bolus of 0.75% ropivacaine [10]. Their study utilized 1 µg/kg fentanyl and did not include PCEA. In contrast, we employed a higher fentanyl concentration (2 µg/mL) with continuous PCEA, yet dexmedetomidine still yielded superior outcomes. In Bajwa’s study, rescue analgesia was required earlier in the fentanyl group (242.16 ± 23.86 minutes) than in the dexmedetomidine group (366.62 ± 24.42 minutes). Similarly, in our study, VAS pain score differences became evident from the 4th postoperative hour (240 minutes) and persisted up to 24 hours. Our methodology, incorporating continuous epidural infusion followed by PCEA, likely contributed to improved pain control and reduced overall drug usage.

Akhondzadeh et al. [14], in a study of 56 patients undergoing femoral neck fracture surgery, found that the dexmedetomidine group had a significantly longer duration of sensory block (311.2 ± 60.3 minutes) compared to the fentanyl group (226.6 ± 46.1 minutes; P = 0.045). The onset times for sensory and motor block were also shorter with dexmedetomidine (3.5 ± 0.6 minutes and 17.5 ± 1.9 minutes, respectively) than with fentanyl (6.0 ± 1.1 minutes and 22.6 ± 2.2 minutes; P < 0.001). VAS scores were lower (4.9 ± 0.6 vs. 5.8 ± 0.9; P < 0.001), and fewer rescue analgesics were required (2.54 ± 1.36 mg vs. 3.15 ± 1.64 mg; P < 0.05). Despite differences in surgical populations, our findings similarly demonstrate the enhanced analgesic profile of dexmedetomidine.

Park et al. advocated for continuous infusion in PCEA for improved pain relief and fewer side effects [11]. Their observations are reflected in our results, no patient in either group experienced bradycardia, hypotension, pruritus, respiratory depression, or urinary retention after catheter removal. Sedation scores remained low, likely due to the steady infusion rate of 5 mL/hr instead of intermittent bolus dosing. Although heart rate was lower in the dexmedetomidine group, the difference was not statistically significant, and no hemodynamic interventions were required. A slightly higher incidence of nausea and vomiting was reported in the fentanyl group compared to dexmedetomidine, though this difference was not statistically significant.

Both groups maintained a Modified Bromage Scale score of 0 throughout the postoperative period, indicating preserved motor function. None of the patients required rescue analgesia, as VAS scores remained below four in both groups. Similarly, Akhondzadeh et al. [14] reported more instances of dry mouth, hypotension, and bradycardia in the dexmedetomidine group, and more nausea and vomiting in the fentanyl group. However, none of these differences were statistically significant, and no respiratory depression was observed.

Thus, dexmedetomidine offers better analgesic quality and reduced drug requirements without increasing the risk of adverse effects. Both adjuvants are safe and effective, but dexmedetomidine appears preferable for enhanced postoperative pain control in patients undergoing total abdominal hysterectomy.

A key strength of our study is its focus on a homogeneous surgical population and the use of PCEA for objective outcome assessment. This design addresses the gap identified by Park et al. in the literature regarding the need for studies incorporating continuous epidural infusions [11]. The combination of general and epidural anesthesia enabled accurate evaluation of postoperative analgesic outcomes.

The primary limitation of our study is the restricted observation period, limited to the first 24 postoperative hours. Additional limitations include the small sample size and the single-center design. A longer follow-up with a larger sample may provide deeper insights, particularly regarding delayed adverse effects or prolonged analgesic efficacy.

## Conclusions

This study found that dexmedetomidine and fentanyl, when used with low concentrations of ropivacaine (0.125%) in epidural blocks, provide effective and prolonged pain relief without significant motor blockade. The PCEA pump offered objective pain assessment, showing that both adjuvants deliver satisfactory analgesia for up to 24 hours. Hemodynamic parameters, including pulse rate and MAP, were similar between the ropivacaine with dexmedetomidine group and the ropivacaine with fentanyl group. Hemodynamic fluctuations were minimal and not significant across both groups. Motor blockade was absent, and sedation levels were minimal, likely due to the absence of bolus dose administration of adjuvants. Overall, both dexmedetomidine and fentanyl proved effective for postoperative pain management, with dexmedetomidine demonstrating a potentially superior quality of analgesia, reduced total ropivacaine consumption, and a comparable hemodynamic safety profile.

## References

[1] Kuthiala G, Chaudhary G (2011). Ropivacaine: a review of its pharmacology and clinical use. Indian J Anaesth.

[2] Patil SS, Kudalkar AG, Tendolkar BA (2018). Comparison of continuous epidural infusion of 0.125% ropivacaine with 1 μg/ml fentanyl versus 0.125% bupivacaine with 1 μg/ml fentanyl for postoperative analgesia in major abdominal surgery. J Anaesthesiol Clin Pharmacol.

[3] Marhofer D, Kettner SC, Marhofer P, Pils S, Weber M, Zeitlinger M (2013). Dexmedetomidine as an adjuvant to ropivacaine prolongs peripheral nerve block: a volunteer study. Br J Anaesth.

[4] Salgado PF, Sabbag AT, Silva PC (2008). Synergistic effect between dexmedetomidine and 0.75% ropivacaine in epidural anesthesia. Rev Assoc Med Bras (1992).

[5] Kaur S, Attri JP, Kaur G, Singh TP (2014). Comparative evaluation of ropivacaine versus dexmedetomidine and ropivacaine in epidural anesthesia in lower limb orthopedic surgeries. Saudi J Anaesth.

[6] Hong JY, Jee YS, Jeong HJ, Song Y, Kil HK (2010). Effects of epidural fentanyl on speed and quality of block for emergency cesarean section in extending continuous epidural labor analgesia using ropivacaine and fentanyl. J Korean Med Sci.

[7] King MJ, Bowden MI, Cooper GM (1990). Epidural fentanyl and 0.5% bupivacaine for elective caesarean section. Anaesthesia.

[8] Cherng CH, Yang CP, Wong CS (2005). Epidural fentanyl speeds the onset of sensory and motor blocks during epidural ropivacaine anesthesia. Anesth Analg.

[9] Gambling DR, Yu P, Cole C, McMorland GH, Palmer L (1988). A comparative study of patient controlled epidural analgesia (PCEA) and continuous infusion epidural analgesia (CIEA) during labour. Can J Anaesth.

[10] Bajwa SJ, Arora V, Kaur J, Singh A, Parmar SS (2011). Comparative evaluation of dexmedetomidine and fentanyl for epidural analgesia in lower limb orthopedic surgeries. Saudi J Anaesth.

[11] Park SJ, Shin S, Kim SH, Kim HW, Kim SH, Do HY, Choi YS (2017). Comparison of dexmedetomidine and fentanyl as an adjuvant to ropivacaine for postoperative epidural analgesia in pediatric orthopedic surgery. Yonsei Med J.

[12] Kiran S, Jinjil K, Tandon U, Kar S (2018). Evaluation of dexmedetomidine and fentanyl as additives to ropivacaine for epidural anesthesia and postoperative analgesia. J Anaesthesiol Clin Pharmacol.

[13] Sarkar A, Bafila NS, Singh RB, Rasheed MA, Choubey S, Arora V (2018). Comparison of epidural bupivacaine and dexmedetomidine with bupivacaine and fentanyl for postoperative pain relief in lower limb orthopedic surgery. Anesth Essays Res.

[14] Akhondzadeh R, Olapour A, Javaherforooshzadeh F, Rashidi M, Bakhtiari N, Hosseininejad F (2023). Dexmedetomidine or fentanyl, which one is better as an adjunct drug in epidural anesthesia and causes more postoperative pain reduction? A comparative study, a randomized clinical trial. Anesth Pain Med.

